# Unexpected modulation of Hna phage defense activity by the symbiotic regulator NolR

**DOI:** 10.1128/jb.00182-25

**Published:** 2025-08-18

**Authors:** Leah M. Sather, Niousha Fazeli, Jason V. S. Kearsley, Kathryn Jones, Turlough M. Finan

**Affiliations:** 1 Department of Biology, McMaster University 98616, Hamilton, Ontario, Canada; 2 Department of Biological Science, Florida State University 123386, Tallahassee, Florida, USA; University of California San Francisco, San Francisco, California, USA

**Keywords:** phage defense, NolR, *Sinorhizobium*, *lpsS*, Hna, gene expression, *nod *genes, transcriptional regulator

## Abstract

The Hna phage defense system is one of many systems that protect bacteria against bacterial viruses (phages). Hna was first discovered in the nitrogen-fixing alphaproteobacterium *Sinorhizobium meliloti*, which forms root nodules on leguminous plants. We report that the efficacy of the Hna system depends on NolR, a transcriptional regulator known to regulate expression of nodulation genes. Strains carrying a mutant *nolR* gene (e.g., the widely used laboratory strain Rm1021) display dramatically reduced Hna-mediated phage resistance compared to those with the wild-type *nolR* gene. *hna* expression is approximately doubled in *nolR^+^* (wild-type) compared to *nolR^–^* strains. Integration of a second copy of *hna* increased phage resistance in a *nolR^–^* strain >1,000-fold, indicating that a moderate *hna* expression difference is sufficient to affect the Hna phage resistance phenotype. NolR does not appear to directly regulate *hna*, as there is no predicted NolR binding site upstream of *hna*, and purified NolR protein does not bind to the *hna* upstream sequence. Other genes whose transcription is regulated by NolR were identified through RNA-seq experiments. These include the lipopolysaccharide sulfotransferase gene *lpsS*, which is located downstream of a NolR binding site. This work illustrates how modest differences in expression between strains can dramatically alter the protective phenotype of a defense system.

**IMPORTANCE:**

The ability of a bacterial culture to survive phage infection is significant in both medical (phage therapy) and industrial (e.g., cheese production) contexts. This study describes a factor that influences the efficacy of a recently discovered phage defense system (Hna) in the agriculturally relevant soil bacterium *Sinorhizobium meliloti*. Like other phage defense systems, Hna systems undergo extensive horizontal transfer and must be able to maintain functionality across different genetic backgrounds. Our work demonstrates that host factor differences can significantly impact the performance of phage defense systems.

## INTRODUCTION

Bacteria employ a diverse array of specialized defense systems to protect themselves against bacteriophage (phage) infection. These systems are often horizontally transferred between different bacteria (1–3). However, a particular defense system will not necessarily be equally effective in different species or even different strains. Little is currently known about how or why the efficacy of a phage defense system may vary across different genetic backgrounds. Differences in system expression, intracellular conditions, or the presence of interacting proteins between hosts could all conceivably alter the level of protection a system provides. Improved understanding of the determinants of defense system success could inform decision-making in phage therapy and bacterial engineering applications, since it may allow better prediction of whether a defense system is likely to be active in a particular strain.

We previously identified a phage resistance gene (*hna*) in the nitrogen-fixing soil bacterium *Sinorhizobium meliloti* (4). *S. meliloti* forms nodules on the roots of leguminous plants, such as alfalfa (*Medicago sativa*) and the model legume *Medicago truncatula*. Its genome consists of a single 3.7 Mb chromosome as well as two extra-chromosomal replicons: pSymA (1.4 Mb) and pSymB (1.7 Mb). Hna is encoded on the pSymA megaplasmid, which also carries many of the *nod* and *nif* genes required for root nodule formation and nitrogen fixation (4, 5). Rm5000 and Rm2011 strains of *S. meliloti* in which pSymA is removed become sensitive to diverse phages, and the re-introduction of *hna* alone is sufficient to restore complete phage resistance (4). Hna homologs are present in isolates that span the bacterial domain, and *hna* appears to be subject to extensive horizontal gene transfer (4).

Hna is a single superfamily II helicase-nuclease protein and confers phage resistance through an abortive infection (Abi) mechanism, meaning that cells carrying the system die upon infection but that no new phages are produced (4). Although the specific molecular mechanism by which Hna prevents phage propagation is still unknown, we have shown that the system’s Abi response may be activated by a phage single-stranded DNA binding protein (SSB) (4).

In the course of characterizing *hna*, we were surprised to see a dramatic influence of the genetic background when we tested the protective phenotype of *hna* in two similar laboratory strains of *S. meliloti* (Rm5000 and Rm1021), both of which were originally derived from the wild-type *S. meliloti* isolate SU47 (6, 7). Hna confers robust phage defense in an Rm5000 background, but is much less protective in an Rm1021 derivative—it reduces plaque formation >10^6^ times in Rm5000, but less than 10 times in Rm1021. Here, we show that *hna*’s lowered efficacy in the Rm1021 background is primarily caused by a frameshift mutation in the chromosomal gene *nolR*. NolR is an ArsR/SmtB family transcriptional regulator and was first described in *S. meliloti* strain AK631 as a repressor of the *nod* genes (8, 9). The *nod* genes encode proteins involved in the synthesis of Nod factor and are required for nodulation of the host plant (9–11). NolR was shown to bind to *nod* gene promoters, and a consensus NolR binding site has been defined (8, 10, 11). Analyses of the proteomes of *nolR*^+^ and *nolR*^–^ strains indicated that NolR may have a more global regulatory scope (12, 13). NolR homologs have also been shown to regulate a variety of symbiotic genes in *Sinorhizobium fredii* and to influence virulence in *Brucella melitensis* (14–16).

Using RNA-seq and reporter gene fusions, we have found that the presence of the wild-type *nolR* gene modestly increases the expression of *hna*. Hna protein levels are also elevated in *nolR*^+^ cells, consistent with the gene’s increased transcription. This NolR-mediated increase in *hna* expression is adequate to explain the improved Hna defense, since a similar improvement in phage resistance phenotype was achieved when a second copy of *hna* was introduced in a *nolR*^–^ background. We have also identified other potential members of the NolR regulon. In particular, our results suggest that the lipopolysaccharide sulfotransferase gene *lpsS* is directly regulated by NolR.

## MATERIALS AND METHODS

### Media and growth conditions

Bacterial strains and plasmids used in this study are listed in Table S1. The *S. meliloti* strains used here are derivatives of the laboratory strains Rm5000 and Rm1021, which were independently derived from *S. meliloti* isolate SU47 (6, 7). All the Rm1021-background strains were constructed in RmP110, an Rm1021 strain in which a frameshift mutation in *pstC* was corrected (17, 18).

*S. meliloti* cultures were grown at 30°C in LB medium (10 g/L tryptone, 5 g/L yeast extract, 5 g/L NaCl) supplemented with 2.5 mM MgSO_4_, 2.5 mM CaCl_2_, and 38 µM FeCl_3_ (LBmc + FeCl_3_). Antibiotics were added when relevant at the concentrations listed in (4), unless otherwise noted. Minimal medium contained 1 × M9 salts, 1 mM MgSO_4_, 0.25 mM CaCl_2_, 38 µM FeCl_3_, 42 nM CoCl_2_, 1 µg/mL biotin, 1.6 µg/mL thiamine hydrochloride, and 10 mM *trans*-4-hydroxy-l-proline. Phage lysates were prepared as described previously (4).

### Phage assays

Plaque assays were conducted using soft agar overlays as described in (4). For phage spot assays, 20 µL of each phage dilution was pipetted onto plates overlaid with soft agar containing 100 µL of bacterial culture (OD_600 _of ~1.1).

### Conjugations and transductions

Triparental conjugations were performed as described in (4).

The *S. meliloti* transducing phage φM12 was used to transduce markers between strains (6). Cultures of donor strains were infected with phage at OD_600_ of ~0.4 and incubated overnight to produce a transducing lysate. Lysates were treated with chloroform (~200 µL) to kill any surviving cells. Five hundred microliters of diluted phage lysate (a 1:10 or 1:25 dilution in LBmc + FeCl_3_ medium) was mixed with 500 µL of overnight recipient culture and incubated at 30°C for approximately 20 min to allow phage adsorption. Cells were then centrifuged, rinsed twice with saline, resuspended in saline, and plated on LB medium with antibiotics to select for transductants. MgSO_4_ and CaCl_2_ were omitted from the selective plates to limit further phage adsorption.

### Tn5 insertion library experiment

To create a Tn5 insertion library, the self-transmissible plasmid pRK602 (a derivative of pRK600 carrying Tn5) was conjugated into *S. meliloti* Rm5000 (Rif^R^) (19). After overnight growth at 30°C, mating spots were suspended in saline and plated on LBmc + FeCl_3_ containing rifampicin, neomycin (100 µg/mL), and streptomycin (100 µg/mL) to select for *S. meliloti* carrying a Tn5 insertion. Tn5 confers streptomycin resistance in *S. meliloti* (20), so streptomycin was included in the medium to limit the growth of spontaneous rifampicin-resistant *Escherichia coli* mutants. After three days of growth, ~3,000 of the resulting colonies were suspended in 1 mL LBmc + FeCl_3_ medium. The pooled colonies were then subcultured to an OD_600_ of ~0.05 in LBmc + FeCl_3_ + Nm + Rif and infected with the transducing phage φM12. The resulting lysate was used to transduce the Tn5 insertion library and flanking regions from Rm5000 into RmP110, selecting for neomycin resistance. After a single round of streak purification, the phage sensitivity of 100 transductants was tested by phage spot assay with phages 5A and 3K, using φM12 as a control for phage sensitivity, as φM12 forms plaques on both *hna^–^* and *hna^+^* lawns*.* Cultures that initially failed to produce a robust lawn were re-tested after a second round of streak purification to dilute out any φM12 virions carried over from the initial transduction.

To determine the location of Tn5, genomic DNA from a phage-resistant transductant was digested with *PstI* and self-ligated. DNA flanking the Tn5 insertion was amplified by PCR using primers specific to the IS50 sequence and then Sanger sequenced (primer sequences: GGGTTCACTCCGTTCTCTTGC and GGTTCCGTTCAGGACGCTAC) (21).

### Construction of *nolR* deletion and correction strains

To delete *nolR*, a derivative of the Gm^R^ *sacB* plasmid pJQ200SK (22) carrying at its SmaI site an 843 bp fragment amplified from upstream of the *nolR* gene (Fragment 1; primer sequences: GGCGGCCGCTCTAGAACTAGTGGATCCCCCGATCATGAGCGAGAAACCC [F] and GGACGTCTTGGGAGACACACGTAGTTATGATTGATGAAG [R]) joined to a 741 bp fragment containing the sequence directly downstream of *nolR* (Fragment 2; primer sequences: TCATAACTACGTGTGTCTCCCAAGACGTCCTTCG [F] and GATAAGCTTGATATCGAATTCCTGCAGCCCGCGGTTGGACATGTGTTTCC [R]) was constructed by Gibson assembly (23). The plasmid was then conjugated into *S. meliloti* strains RmP110, RmP4323 (Rm5000 ΔpSymA with pTH1937 carrying *hna* integrated at Δ*hypRE*::FRT) (4), and RmP6008 (RmP110 ΔpSymA φRmP4323), and transconjugants were selected on LBmc + FeCl_3 _+ Gm + Sm (for RmP110 background strains) or Rif (for Rm5000 background strains).

Individual transconjugants were picked and grown in liquid medium without antibiotics and then plated on medium containing 5% sucrose to select for cells that had lost the *sacB* marker. Colonies were then screened for loss of Gm resistance. Deletion of *nolR* was checked by PCR amplification across the deleted region, using the Fragment 1 F and Fragment 2 R primers described above, which yields a shorter product if *nolR* is deleted.

The RmP110 *nolR* frameshift was corrected in an analogous manner to the deletion of *nolR*. The wild-type *nolR* gene, along with its upstream and downstream sequence, was amplified from Rm5000 DNA using the Fragment 1 forward primer and the Fragment 2 reverse primer used above and cloned into pJQ200SK via Gibson assembly (22, 23). The plasmid was conjugated into *S. meliloti* RmP110 and RmP6008, and double crossover mutants were identified as described above. Correction of the *nolR* mutation was confirmed by Sanger sequencing of PCR products amplified across the *nolR* gene.

### Complementation of *nolR* deletion

The *nolR* gene, including 184 bp upstream and 15 bp downstream sequence, was amplified from Rm5000 DNA and cloned into the EcoRV site in the *S. meliloti* integrative plasmid pTH1937 by Gibson assembly (23, 24). pTH1937 carries an FRT site, which allows integration of the plasmid at an FRT site in the genome (24). The pTH3546 (pTH1937 with *nolR* from Rm5000) plasmid was then conjugated into RmP4253, a ΔpSymA derivative of RmP110 that carries an FRT site at *hypRE* on pSymB, as well as the Flp recombinase expression plasmid pTH2505 (25). The conjugation was performed in the presence of 2.5 mM protocatechuic acid to induce expression of the Flp recombinase and allow integration of the *nolR* plasmid at the pSymB FRT site. The integrated *nolR* plasmid was then transduced into RmP110, RmP6053 (RmP110 Δ*nolR*), and RmP6058 (RmP110 *nolR^+^*). The empty pTH1937 plasmid was also introduced in the same manner to create non-complemented control strains. Transductants were unable to grow on minimal medium with *trans*-4-hydroxy-l-proline as a sole carbon source, indicating that the plasmids had integrated at *hypRE* as expected (26).

### Introduction of a second copy of Hna

To create strains carrying two copies of *hna*, the *hna* plasmid pTH3258 (pTH1937 with *hna*) integrated at Δ*hypRE::*FRT in RmP4323 (4) was transduced into RmP6053 (RmP110 Δ*nolR*) and RmP6058 (RmP110 *nolR^+^*), which carry the native copy of *hna* on pSymA. The empty pTH1937 plasmid was also transduced from RmP4321 (4) into both recipient strains to create control strains carrying only a single copy of *hna*.

### RNA-seq and differential expression analysis

Bacterial RNA was isolated based on the hot phenol method described in (27). Cultures of RmP6053 (RmP110 Δ*nolR*) and RmP6058 (RmP110 *nolR^+^*) were grown in triplicate to OD_600 _of ~0.6 in LBmc + FeCl_3_ medium. Upon reaching the desired density, 4.5 mL of culture was added to 500 µL ice-cold stop solution (5% unbuffered phenol in ethanol) and kept on ice until centrifugation. The samples were then centrifuged (5,020 × *g* for 17 min at 4°C) and the cell pellets were stored at −80°C until further processing.

Prior to RNA extraction, each pellet was resuspended in 300 µL resuspension solution (10 µL β-mercaptoethanol/mL dH_2_O). The cell resuspensions were then mixed with an equal volume of the hot phenol solution described in (27), vortexed for 30 s, and boiled for 1 min in a 95°C water bath. The lysates were centrifuged (16,110 × *g* for 10 min at 4°C), and the supernatants were extracted twice with phenol:chloroform and once with chloroform alone prior to isopropanol precipitation over several days at −20°C. The resulting nucleic acids were then rinsed twice with 70% ethanol and finally resuspended in 50 µL DEPC-treated dH_2_O.

Initially, each sample was incubated at 37°C in 100 µL reactions containing 3 U DNase I-XT (New England Biolabs) to degrade co-purified DNA. After 1.5 h, an additional 3 U DNase I-XT was added, and samples were incubated for another 1.5 h. The RNA was then re-extracted once with phenol:chloroform and once with chloroform alone and re-precipitated and rinsed as before. To check for the presence of contaminating DNA, PCR with *S. meliloti* chromosomal primers (Fragment 2 F and R primers described above) was performed on 600–900 ng of each RNA sample. RNA concentrations were estimated using a NanoDrop spectrophotometer. Three of the six reactions yielded a PCR product after 35 cycles, so all samples were re-treated with 8 U DNase for 4 h at 37°C and then re-extracted as described above. After the second round of DNase treatment, none of the samples produced a PCR product. The RNA was also visualized on an agarose gel to confirm that it had not been extensively degraded. Three distinct bands, assumed to correspond to 23S, 16S, and 5S rRNA, were visible for each sample.

rRNA depletion, cDNA library preparation, and Illumina sequencing (NovaSeq PE100) were performed at the Centre d’Expertise et de Services Genome Québec. The quality of the resulting sequencing reads was checked with FastQC (v 0.12.1) (28). Reads were trimmed with Trimmomatic (v. 0.39) using the suggested paired-end settings (provided NovaSeq adapter sequences: AGATCGGAAGAGCACACGTCTGAACTCCAGTCAC and AGATCGGAAGAGCGTCGTGTAGGGAAAGAGTGT) (29). The trimmed reads were then mapped to an *S. meliloti* 1021 reference sequence using Bowtie2 (v 2.2.2) with default settings (30). Counts of reads aligning to each coding sequence (CDS) feature were quantified using htseq-count in stranded (reverse) nonunique union mode (31). Differential gene expression analysis was performed using the DESeq2 R package (32).

### β-glucuronidase activity assay

The gentamicin-resistant integrated *gusA* reporter constructs were transduced from the original fusion library strains (FL591, FL6084, and FL5430) (33) into RmP110, RmP6053, and RmP6058. Assays were conducted in 96-well plates based on the protocol described by Cowie et al. (33). Cells were harvested from liquid culture after overnight growth, rinsed, resuspended in saline, and diluted to an OD_600 _<0.5. Twenty microliters of cell suspension was mixed with 80 µL of modified Gus buffer (50 mM sodium phosphate buffer [pH 7.0], 1 mM EDTA [pH 7.0], 0.1% Triton X-100, 40 mM β-mercaptoethanol, and 0.44 mg/mL 4-nitrophenyl-β-D-glucuronic acid) and incubated at 30°C until a sufficient yellow color developed. Reactions were stopped by the addition of 100 µL 1 M Na_2_CO_3_. Absorbance at 405 nm was measured using a Cytation 3 plate reader. Miller units for β-glucuronidase activity were calculated using the equation from (33).

### Western blot

In an RmP110 ΔpSymA background (RmP4247), *nolR* was corrected and deleted to generate strains RmP6226 (RmP4247 *nolR*^+^) and RmP6225 (RmP4247 Δ*nolR*), respectively, as described above. To allow detection of Hna levels by Western blotting, a His-tagged copy of *hna* was introduced into these strains at *hypRE* by transduction of neomycin resistance from RmP4510 (4).

Cells (equivalent to 2 mL of OD_600 _of ~2) were harvested after overnight growth, rinsed with 1 mL PBS, and resuspended in 75 µL PBS. After the addition of Laemmli buffer, samples were vortexed, boiled for 15 min, and then stored at −20°C until needed.

Samples were centrifuged for 15 min at 16,060 × *g,* and supernatants were loaded on two SDS-polyacrylamide gels. After electrophoresis, one gel was stained with Coomassie blue to reveal total soluble protein content, while the other was used for Western blotting. For Western blotting, resolved proteins were electro-transferred from the gel to a PVDF membrane using a semi-dry apparatus. The membrane was blocked in 5% skim milk powder (2 h to overnight) and then hybridized with mouse anti-His tag primary antibody (BioRad), followed by a goat anti-mouse-alkaline phosphatase conjugated secondary antibody (BioRad). The membrane was rinsed between steps with TBST (1× TBS, 0.05% Tween-20) for 3 × 10 min. The secondary antibody was detected colorimetrically using NBT/BCIP in 1× AP buffer (20 mM Tris-HCl [pH 9.0], 20 mM NaCl, 1 mM MgCl_2_).

### Purification of the NolR protein 

*nolR* with a C-terminal 6× His tag (sequence: GGGHHHHHH) was cloned into the NcoI site in pET28a (Novagen) by Gibson assembly (23). The resulting construct was transformed into BL21 for overexpression. A culture of BL21 carrying the *nolR-*His expression plasmid was grown in 500 mL LB (with 20 μg/mL kanamycin) to OD_600_ of ~0.4, whereupon 1 mM IPTG was added to induce *nolR* expression. Following 3.5 h of additional growth at 30°C, cells were harvested by centrifugation (5,020 × *g* for 20 min at 4°C), rinsed with 50 mL PBS, and then stored as cell pellets at −20°C.

Pellets were thawed and resuspended in 25 mL HisTrap binding buffer (40 mM sodium phosphate, monobasic; 300 mM NaCl; 30 mM imidazole) with 1.5 mM PMSF and then lysed using a French press. The resulting lysate was centrifuged (30 min at 10,000 × *g*) and filtered to remove cell debris. NolR protein was purified using the ÄKTA Start FPLC loaded with a 1 mL HisTrap HP column (Cytiva). Fractions were eluted by increasing the concentration of elution buffer (40 mM sodium phosphate, monobasic; 300 mM NaCl; 500 mM imidazole) over 15 column volumes. The protein contents of eluted fractions were visualized by SDS-PAGE and Coomassie blue staining. The four fractions containing the most NolR protein were pooled together and diafiltered four times through an Amicon Ultra-4 centrifugal filter tube (3 kDa MWCO) to concentrate the protein stock and change its buffer composition. Between rounds of diafiltration, the retained solution was diluted with 2× protein storage buffer (40 mM Tris-HCl [pH 7.5], 200 mM NaCl, 2 mM dithiothreitol), leaving a final imidazole concentration of ~1 mM in the purified protein preparation. The final protein concentration was determined by Bradford assay. The purified protein was diluted 1:1 with glycerol and then flash frozen in liquid nitrogen and stored at −80°C.

### Electrophoretic mobility shift assays (EMSAs)

Fluorescently labeled EMSA probes were generated by PCR using one 5′ 6-FAM-labeled primer and one unlabeled primer. DNA probe (2–4 nM) was added to EMSA binding reactions (10 µL total volume) containing 1× EMSA binding buffer (10 mM Tris-HCl [pH 8.0], 1 mM EDTA [pH 8.0], 50 mM KCl, 5 mM MgCl_2_, 1 mM dithiothreitol, 5% glycerol, 10 µg/mL BSA), 200 µg/mL sonicated salmon sperm DNA, and varying amounts of NolR protein. Mixtures were incubated at room temperature for 30 min before electrophoresis through a native 5% polyacrylamide gel (1× TAE, 0.25% glycerol). Gels were imaged with a Typhoon FLA 9500 imager (GE Healthcare).

EMSA probe sequences are specified in Table S2.

### Localization of predicted NolR binding motifs

The positions of NolR binding motifs were identified using Sequence Searcher (Viral Bioinformatics Research Centre). A custom Python script was developed using Biopython to determine which of the differentially expressed genes have a binding site within 300 bp upstream of their start codon (34).

## RESULTS

### Mutation of the *nolR* transcriptional regulator gene influences the efficacy of *hna*-mediated phage defense

**Fig 1:**
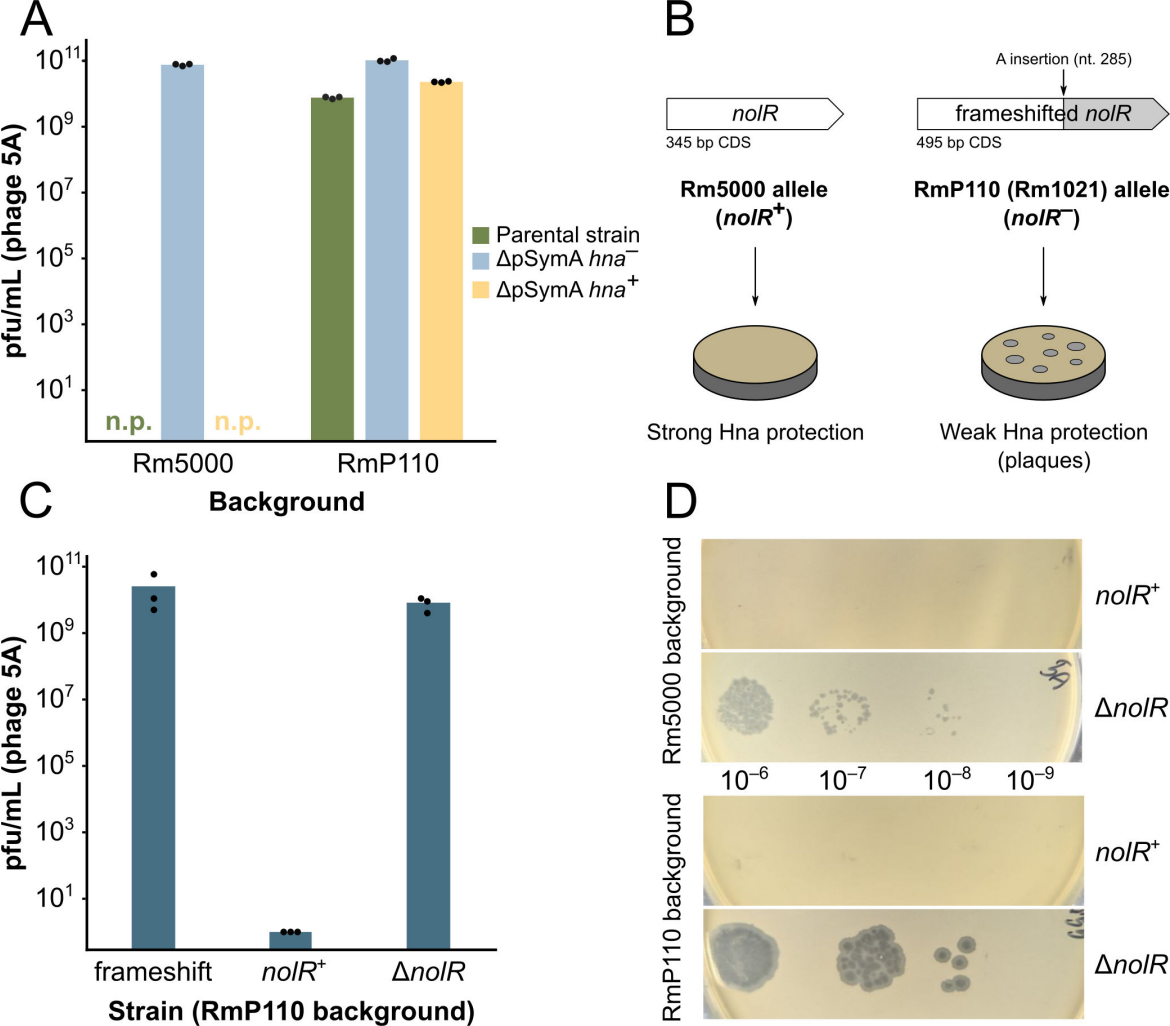
A functional copy of *nolR* dramatically improves the efficacy of *hna-*mediated phage defense. (**A**) Plaque formation of phage 5A on Rm5000 vs. RmP110 (Rm1021) derivatives. Bars represent the mean plaque-forming units (pfu)/mL calculated from plating on three separate cultures. Points represent individual replicates. Parental strain (green) = Rm5000 or RmP110, with its native copy of *hna* on pSymA. ΔpSymA *hna^–^* (blue) = RmP4321 (Rm5000 background) or RmP6007 (RmP110 background). ΔpSymA *hna^+^* (yellow) = RmP4323 (Rm5000 background) or RmP6008 (RmP110 background). “n.p.” indicates that no plaques were observed even when ~8 × 10^7^ plaque-forming units were plated. Strains: Rm5000 (pSymA *hna*^+^), RmP4321 (Rm5000 ΔpSymA Δ*hypRE hna^–^*), RmP4323 (Rm5000 ΔpSymA Δ*hypRE:: hna^+^*); RmP110 (pSymA *hna*^+^), RmP6007 (RmP110 ΔpSymA Δ*hypRE hna^–^*), and RmP6008 (RmP110 ΔpSymA Δ*hypRE:: hna^+^*). (**B**) Schematic depicting the *nolR* alleles in Rm5000 and Rm1021 (RmP110) and their effect on Hna-mediated phage resistance. The position of the frameshift mutation is noted relative to the MQPL start position. (**C**) Plaque formation of phage 5A on WT RmP110 (with a frameshift mutation in *nolR*), RmP110 with the *nolR* frameshift corrected (*nolR^+^*: RmP6058), and RmP110 with *nolR* deleted (Δ*nolR:* RmP6053). All strains carry the native copy of *hna* on pSymA. Bars represent the mean pfu/mL calculated from plating on three separate cultures. Points represent individual replicates. (**D**) Comparison of phage 5A plaquing when *nolR* is deleted in an Rm5000 vs. RmP110 (Rm1021) background. All strains lack pSymA and carry an integrated copy of *hna* at *hypRE* on pSymB. 20 µL of each indicated ten-fold dilution of the phage lysate was spotted onto a bacterial lawn. All images are equally scaled. Strains are as follows: Rm5000 background: *nolR^+^*: RmP4323, Δ*nolR*: RmP6057; RmP110 background: *nolR+:* RmP6060, Δ*nolR:* RmP6055.

Our prior characterization of the *S. meliloti* Hna phage defense system was conducted using two distinct phages: 5A (a clear-plaquing variant of phage vB_SmeP_3T) and 3K (phage vB_SmeM_3K) (4). Phages 5A and 3K are similar to the *E. coli* phages T7 and T4, respectively. In an Rm5000 background, *hna* demonstrates a robust protective effect, reducing plaquing efficiencies at least 10^8^- and 10^6^-fold, respectively, for phages 5A and 3K (4). However, in an Rm1021 (RmP110) background (RmP110 is a *pstC^+^* derivative of Rm1021 in which a frameshift mutation in the gene has been corrected [17]), *hna* only reduces plaquing efficiency by ~5 times compared to a strain lacking the system (Fig. 1A). A similar effect was seen for phage 3K (data not shown), indicating that the strain-dependent difference in resistance affects both very different phages, although in this work, we primarily show results for phage 5A. This difference in the efficacy of the Hna system was unexpected as Rm5000 (rifampicin-resistant) and Rm1021 (streptomycin-resistant) are both spontaneous antibiotic-resistant derivatives of *S. meliloti* isolate SU47 (6, 7). In additional experiments, we tested Rm2011, an SU47 strain that is maintained in numerous laboratory collections and found that strain Rm2011 exhibited the same *hna*-dependent phage resistance phenotype as Rm5000. It therefore appeared likely that the attenuated *hna* phage resistance phenotype of RmP110 results from a mutation acquired in strain Rm1021 or later in RmP110.

To determine the genetic basis for the difference in the phage resistance phenotype between Rm5000 (“strong” phage protection) and RmP110 (“weak” phage protection), we sought to identify a transposon insertion that was linked to the responsible locus (see Materials and Methods). We opted for this approach because previous DNA sequence analysis of Rm1021 and Rm2011 revealed numerous differences (>100 polymorphisms), making it unfeasible to investigate the potential involvement of each mutation individually (35, 36). Accordingly, we generated a random Tn5 insertion library in Rm5000 and transduced neomycin resistance from that library into RmP110. We then screened the transductants for the strong phage protection phenotype. When neomycin resistance (Tn5) from one such phage-resistant colony was transduced into RmP110, 80% of the resulting RmP110 transductants had the strong phage resistance phenotype. This confirmed a close linkage (~14 kb) between the transposon and the relevant locus (19, 37). The DNA sequence of the Tn5 insertion site revealed it was within the *dppA2* gene on the *S. meliloti* chromosome. *dppA2* is located 12 kb from *nolR* (*smc07253*), and since previous studies by Kondorosi and colleagues had shown that the Rm1021 *nolR* gene carries an inactivating frameshift mutation (9), we PCR amplified and sequenced the *nolR* region from Rm5000 and representative transductant colonies. Transductants that had gained the strong phage resistance phenotype had the wild-type *nolR* sequence, while those with the weak phage resistance phenotype carried the frameshift mutation (Fig. 1B). The frameshift mutation consists of a single adenine insertion at CDS position 311 relative to the first nucleotide of the MNRF start position (38) (position 285 relative to the MQPL start—n.b. *nolR* has multiple potential translation start sites predicted by nucleotide sequence analysis [9]).

To confirm that the mutation in *nolR* is responsible for the weak phage resistance phenotype, we corrected the frameshift in RmP110 by recombination with the wild-type allele (see Materials and Methods). The resulting strain displayed drastically improved resistance to both phage 5A (Fig. 1C) and phage 3K (data not shown). We also deleted the *nolR* gene in RmP110 and observed similar phage sensitivity as seen with the frameshifted copy (Fig. 1C).

To check that the effect of *nolR* on *hna* efficacy is not dependent on other loci in the Rm1021 background, we deleted the *nolR* gene in an Rm5000 background (Rm5000 ΔpSymA *hna*^+^ strain RmP4323). As in the Rm1021 background, the Rm5000 *ΔnolR* strain became much more phage-sensitive than the corresponding strain with the wild-type *nolR* sequence (Fig. 1D). Since the deletion of *nolR* conferred a dramatic reduction in phage resistance in both strains, we concluded that the *nolR* mutation in RmP110 (Rm1021) is the main determinant of the difference in *hna*-mediated phage resistance between the two genetic backgrounds. There may be additional factors that affect Hna’s efficiency between the two backgrounds, as the plaques were noticeably smaller when *nolR* was deleted in the Rm5000 background compared to the Rm1021 background (Fig. 1D). We also noticed that the phage 5A plaques were smaller (diameter of ~1 mm vs. ~3 mm) and approximately three times less numerous on the RmP110 strain (i.e., pSymA^+^) compared to an RmP110 ΔpSymA strain in which *hna* was integrated into pSymB at *hypRE* (Fig. 1A), although we did not observe this effect in the Rm5000 background. However, these differences are minor compared to the effect of *nolR*, so we do not investigate these factors any further here. 

### *hna* expression is upregulated in the presence of a functional *nolR* gene

Given the suspected global regulatory role of NolR (12–14), we wondered whether NolR regulates the expression of *hna* or a cooperating protein. To assess NolR’s effect on gene expression, we examined the transcriptome by RNA-seq on RmP110-derived strains with and without a wild-type copy of the gene (*nolR*^+^ vs. Δ*nolR*) and looked for differentially expressed genes. One hundred seven genes were significantly differentially expressed (|log2 fold change| > 1 and adjusted *P* value < 0.05) in the *nolR*^+^ vs. the Δ*nolR* strain (Table 1; Table S3; Fig. S1), excluding *nolR* itself. Genes with significantly decreased expression in the presence of *nolR* (60 total) included the *nod* genes, as expected (8, 9, 11). Other genes included *lpsS*, which encodes a lipopolysaccharide-modifying sulfotransferase (39), *cyaF1*, *exoX*, and the biotin transporter gene *bioB* and its two downstream genes (*smc00965* and *smc00966*). Genes whose expression increased in the *nolR*^+^ strain (47 total) included *smc04246*, which encodes a putative RcnB-family transmembrane protein, several *nap* (nitrate assimilatory pathway) genes, and the predicted diguanylate cyclase gene *smc01464*. The majority of the genes found to be significantly differentially expressed had also shown differential expression in a pilot RNA-seq experiment conducted using two different *nolR^+^* and *nolR^–^* strains (see Table S4 and Note S1), which increased our confidence in the reliability of the results.

**TABLE 1:** Selected genes showing a >2-fold change in expression in the *nolR*^+^ strain relative to the Δ*nolR* strain

Gene^*a*^	LFC^*b*^	Adjusted *P* value	Product description
Downregulated genes/putative operons			
***lpsS***	−3.612	5.69E-102	Stf0 family sulfotransferase
***bioB***	−3.283	1.83E-185	Biotin transporter BioY
*SMc00965*	−2.606	1.05E-115	AMP-binding protein
*SMc00966*	−2.197	5.05E-67	Thiolase family protein
*cyaF1*	−2.934	8.91E-97	Adenylate/guanylate cyclase domain-containing protein
*SMc00878*	−2.072	1.26E-75	LuxR family transcriptional regulator
***nodA***	−1.731	2.89E-31	Nodulation N-acyltransferase NodA
*nodB*	−1.520	1.71E-23	Chitooligosaccharide deacetylase NodB
*nodC*	−1.481	8.52E-29	Chitooligosaccharide synthase NodC
*nodI*	−1.153	4.61E-14	Nodulation factor ABC transporter ATP-binding protein NodI
*nodJ*	−0.808	1.40E-05	ABC transporter permease
***SMc04280***	−1.631	4.66E-31	TadE/TadG family type IV pilus assembly protein
***nodD2***	−1.611	3.18E-32	Transcriptional regulator NodD2
***nodM***	−1.503	1.91E-09	Glutamine–fructose-6-phosphate transaminase (isomerizing)
*nolF*	−1.403	1.68E-17	Nodulation protein NolF
*nolG*	−0.756	1.99E-08	Nodulation protein NolG
*nodN*	NA	NA	
*mcpT*	−1.356	5.93E-33	Methyl-accepting chemotaxis protein
*SMc01718*	−1.150	1.99E-16	TVP38/TMEM64 family protein
*merA1*	−0.616	1.71E-04	NAD(*P*)/FAD-dependent oxidoreductase
*SMc01716*	NA	NA	
***nodD1***	−1.316	3.99E-24	Transcriptional regulator NodD1
*exoX*	−1.180	1.88E-21	Exopolysaccharide production repressor ExoX
***mcpW***	−1.168	5.51E-15	Methyl-accepting chemotaxis protein
*cheW2*	−1.048	2.52E-15	Chemotaxis protein CheW
*mcpV*	−1.151	1.95E-14	Methyl-accepting chemotaxis protein
*mcpU*	−1.092	9.52E-17	Methyl-accepting chemotaxis protein McpU
*phrR*	−1.050	1.54E-21	Helix-turn-helix domain-containing protein
Upregulated genes/putative operons			
*SMc04246*	2.326	4.40E-82	RcnB family protein
***napE***	1.682	6.54E-04	Periplasmic nitrate reductase, NapE protein
*napF*	1.451	6.76E-09	Ferredoxin-type protein NapF
*napD*	1.497	8.28E-04	Chaperone NapD
*napA*	1.490	3.19E-22	Periplasmic nitrate reductase subunit alpha
*napB*	1.281	3.74E-05	Nitrate reductase cytochrome c-type subunit
*napC*	1.654	7.07E-16	Cytochrome c3 family protein
*iatP*	1.566	5.45E-22	ABC transporter permease
*iatA*	1.722	4.30E-27	Sugar ABC transporter ATP-binding protein
*ibpA*	1.687	5.10E-26	Sugar ABC transporter substrate-binding protein
*iolC*	1.245	2.11E-17	IolC (inositol catabolism)
*iolD*	1.192	7.44E-14	IolD
*iolE*	1.195	1.56E-12	IolE
*iolB*	1.117	3.94E-11	IolB
*SMc00431*	1.301	4.24E-12	GFA family protein
*iolA*	1.209	1.27E-12	CoA-acylating methylmalonate-semialdehyde dehydrogenase
*SMc01464*	1.154	8.81E-15	GGDEF domain-containing protein (diguanylate cyclase)
*SMc02868*	1.079	3.92E-20	Efflux RND transporter periplasmic adaptor subunit
*SMc02867*	1.042	9.16E-20	Efflux RND transporter permease subunit
*smeR*	1.024	3.15E-15	TetR family transcriptional regulator
*SMa2245*	1.075	1.80E-16	Hna (DEAD/DEAH box helicase)
*SMa2243*	1.041	9.93E-14	Three-Cys-motif partner protein TcmP

^*a*^ Genes located within 300 bp downstream of a predicted NolR binding site are indicated in bold. Putative transcriptional units are grouped together and separated by shading, with the first gene in the potential operon listed first. NA, not applicable.

^*b*^ LFC: log2-fold change.

**Fig 2:**
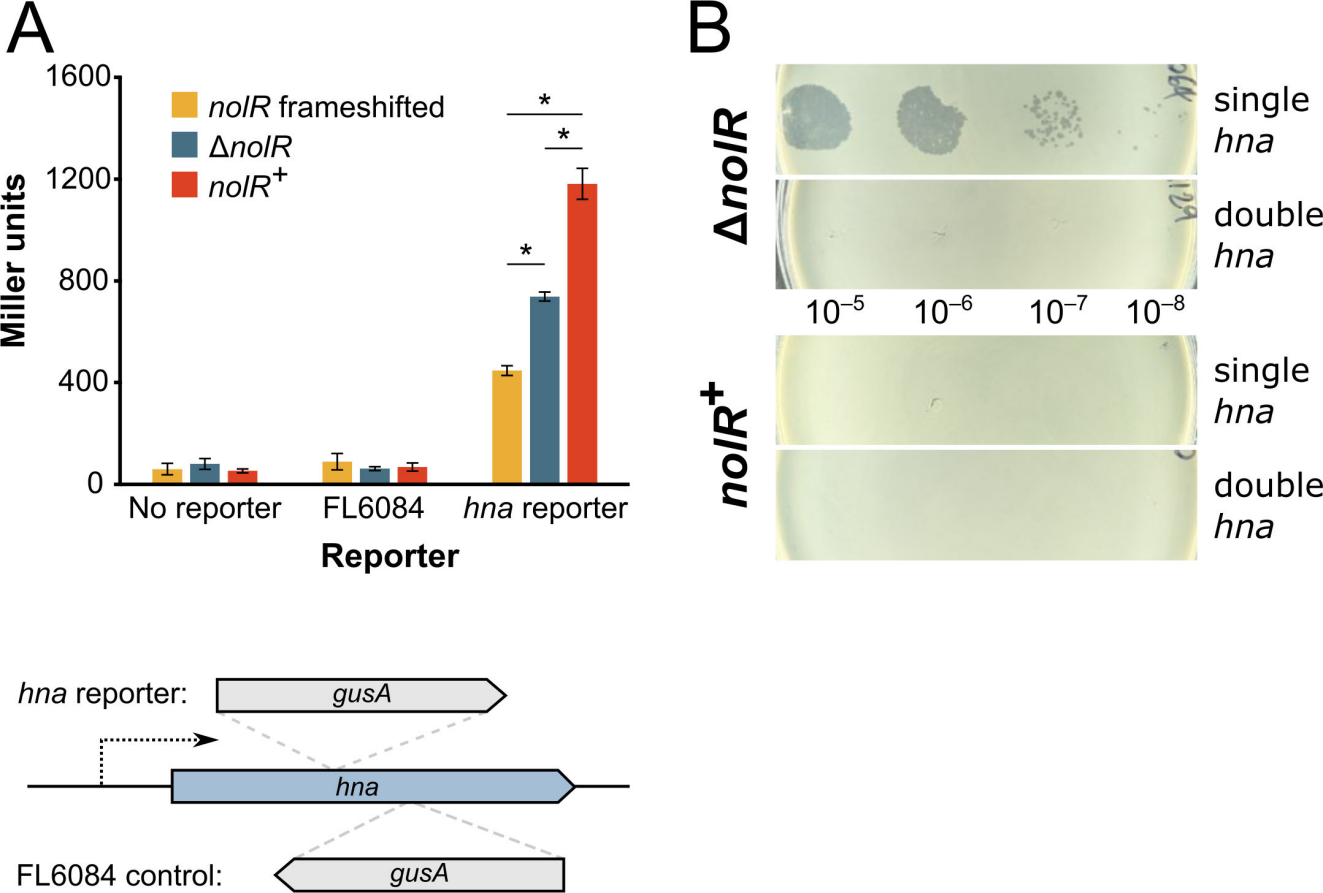
Increased *hna* expression in the presence of functional NolR corresponds with improved *hna* phage defense. (**A**) β-glucuronidase activity from a *gusA* fusion to the *hna* promoter in RmP110 strains in which *nolR* is frameshifted, entirely deleted, or where the frameshift mutation is corrected. Background β-glucuronidase activity was measured in the parental strains without a *gusA* reporter construct, as well as in strains carrying the FL6084 reporter fusion, which is integrated at the end of the *hna* gene and in the opposite orientation of *hna* transcription. Bars represent the means of three biological replicates. Error bars indicate means +/− SD. Asterisks indicate statistically significant differences between samples tested by one-way ANOVA followed by Tukey HSD tests (all adjusted *P* values < 0.005). The schematic indicates the position of the *gusA* reporter gene relative to *hna* in the *hna* and control reporter strains. (**B**) Comparison of phage 5A plaquing in RmP110 strains with and without *nolR* with one or two copies of *hna*. All strains carry the native *hna* on pSymA. Strains with two *hna* copies also have a copy of *hna* at *hypRE* on pSymB. 20 µL of each indicated ten-fold dilution of the phage lysate was spotted onto a bacterial lawn. All images are equally scaled. Strains are as follows: Δ*nolR:* RmP6064 (single *hna*), RmP6129 (double *hna*); *nolR^+^:* RmP6066 (single *hna*), RmP6130 (double *hna*).

We observed a moderate (~2 times) but statistically significant increase in the expression of *hna* in the *nolR*^+^ strain (Table 1). To examine this expression difference further, we used an integrated *hna-gusA* reporter gene fusion (33), and we saw a similar effect of *nolR* on *hna* expression in RmP110 strains in which the *nolR* gene sequence had been either corrected or deleted (Fig. 2A). An integrated reporter construct (FL6084) in which *gusA* was in the opposite orientation to the *hna* gene was included to measure the background reporter signal. The *hna* reporter strain with the corrected *nolR* gene sequence displayed ~1.6 times higher β-glucuronidase activity than the *nolR* deletion strain (Fig. 2A). Interestingly, the strain carrying the frameshifted copy of *nolR* showed significantly lower reporter activity than the Δ*nolR* strain. The *S. meliloti* NolR protein sequence is very similar to the *Sinorhizobium fredii* NolR (~90% amino acid identity), for which the crystal structure has been solved (10). Based on an alignment of the two protein sequences, as well as an Alphafold structure prediction, the frameshift in the Rm1021 NolR occurs within the final alpha helix (α5), outside of the helix-turn-helix motif (10, 38, 40, 41). This helix is not directly involved in DNA binding and may instead play a role in dimerization (10, 38). Therefore, it is possible that the mutant NolR may possess some altered activity that could explain the discrepancy between RmP110 and the deletion mutant.

### Provision of a second copy of *hna* improves phage resistance in a *nolR*^–^ strain

**Fig 3:**
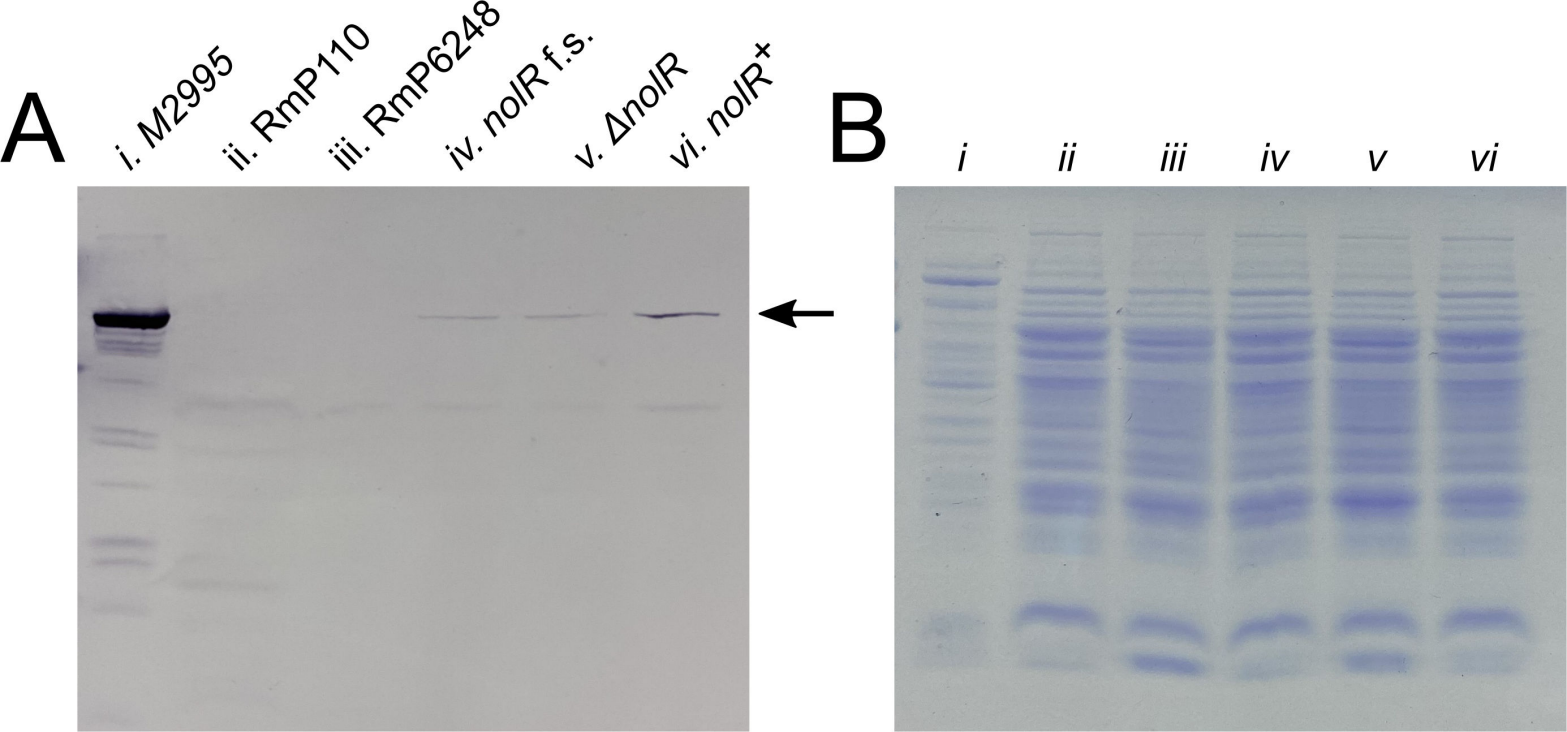
Increased Hna protein level in *nolR^+^* strains. (A) Representative Western blot detecting His-tagged Hna in *nolR^–^* vs. *nolR^+^* strains, performed with anti-His primary antibody. The position of His-tagged Hna protein (~95 kDa) is indicated with an arrow. Lane i: Culture of M2995 (*E. coli* carrying the Hna-His expression plasmid pTH3562) in which *hna* expression was induced for 3 h. Lane ii: RmP110 control (no His-tagged Hna). Lane iii: RmP6248 control (no Hna). Lane iv: RmP6199 (RmP110 ΔpSymA frameshifted *nolR* with His-tagged Hna). Lane v: RmP6231 (RmP110 ΔpSymA Δ*nolR* with His-tagged Hna). Lane vi: RmP6233 (RmP110 ΔpSymA *nolR^+^* with His-tagged Hna). (B) Aliquots of the same samples run on a second SDS-polyacrylamide gel and stained with Coomassie blue to confirm approximately equivalent total soluble protein content across all samples.

To determine whether such a modest increase in *hna* expression could explain the dramatic improvement of *hna* anti-phage activity in a *nolR*^+^ strain, we constructed *hna*-diploid strains in *nolR^–^* and *nolR^+^* backgrounds. The second copy of *hna* with its native promoter region was integrated into an FRT site at the *hypRE* locus on pSymB (see reference 4) in a strain containing the native copy of *hna* on pSymA. We reasoned that the presence of two copies of *hna* in a *nolR*^–^ strain should approximately double *hna* expression, mimicking the *hna* transcription level found in a *nolR*^+^ background. The provision of a second copy of *hna* reduced phage 5A plaque formation >10^3^-fold (Fig. 2B) and reduced phage 3K plaque formation ~100-fold (not shown), compared to the *nolR*^–^ strain carrying only a single *hna* gene, suggesting that doubling *hna* expression is indeed sufficient to affect the *hna* phage resistance phenotype. For unknown reasons, the reduction in plaque formation, while dramatic, was not equivalent to the extent of reduction seen in the presence of a functional *nolR* (see Fig. 1C). We also created an *hna* expression plasmid by inserting *hna* downstream of the P_tac_ promoter in pTH1227, which displays some constitutive, leaky expression in *S. meliloti* (42, 43). We observed >10^6^-fold reductions in 5A plaque formation in the absence of IPTG inducer when the *hna* plasmid was provided in a *nolR^–^* strain (Table S5), independently confirming that increased *hna* expression improves phage defense. When we compared the amount of Hna protein present in *nolR*^–^ and *nolR*^+^ cultures by Western blot using strains carrying integrated C-terminally His-tagged Hna, we observed increased intensity of the Hna-His band in the *nolR*^+^ vs. the *nolR*^–^ samples (Fig. 3). We conclude that *nolR* influences the *hna* phage resistance phenotype due to its effect on the expression of *hna* and that increased *hna* transcription in a *nolR*^+^ strain leads to an increase in cellular Hna levels and ultimately to better phage protection.

### NolR does not bind the *hna* upstream region *in vitro.*

**Fig 4:**
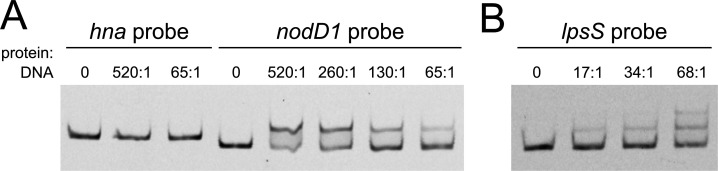
Electrophoretic mobility shift assays with purified NolR protein. (**A**) Varied amounts of purified NolR-His protein added to 6-FAM-labeled DNA probe encompassing the *hna* start codon and 280 bp of upstream sequence (no shift) and to a 6-FAM-labeled probe containing first base of the *nodD1* CDS and 240 bp of upstream sequence. The *nodD1* probe sequence includes a known NolR binding motif. Numbers above each lane indicate the approximate ratio of NolR monomers:DNA probe. Each binding reaction contains ~0.02 pmol DNA probe. (**B**) Varied amounts of purified NolR-His protein added to 6-FAM-labeled probe that includes the *lpsS* start codon and 207 bp of upstream sequence. Numbers above each lane indicate the approximate ratio of NolR monomers:DNA probe. Each binding reaction contains ~0.038 pmol DNA probe. For both gels in panels A and B, samples were incubated at room temperature for 30 min and then run on a 5% polyacrylamide gel for 2 h at 50 V. Image contrast and brightness were adjusted to improve visibility of bands.

A consensus sequence [(A/T)TTAG-N (9)-A(T/A)] for the NolR binding site has been defined (8, 10, 11). Examination of the DNA upstream of *hna* did not reveal a predicted NolR binding sequence, suggesting that *hna* is not directly regulated by NolR. To verify that NolR does not bind to an as-of-yet unknown alternate binding site in the region upstream of *hna*, we conducted electromobility shift assays (EMSAs) using purified C-terminally His-tagged NolR protein and a 283 bp DNA probe encompassing the *hna* start codon and 280 bp of *hna* upstream sequence. This sequence contains a putative modified RpoD (σ^70^) binding sequence that was predicted between 10 and 35 nt upstream of the gene’s transcriptional start site (Fig. S2) (35, 44). A probe containing the known NolR binding site upstream of the *nodD1* gene was used as a positive control for protein binding. The purified NolR protein demonstrated binding to the *nodD1* upstream probe (Fig. 4A). In contrast, we did not observe any shift even when an equivalent amount of protein was added to the *hna* upstream probe, consistent with the lack of a NolR binding site within the sequence (Fig. 4A).

We were also curious whether direct regulation by NolR could explain the differential expression of any of the genes from our RNA-seq experiment. We searched for occurrences of the Cren et al. (11) motif (A/T)TTAG-N (9)-A(T/A), as well as the more stringent Lee et al. (10) motif (A/T)TTAG-N (8)-GA(T/A)G across the *S. meliloti* genome (Table S6). *S. meliloti* Rm1021 contains 308 predicted Cren et al. (11) binding motifs (148 on the chromosome, 88 on pSymA, and 72 on pSymB). Only thirty of these sites (16 chromosomal, 10 on pSymA, and 4 on pSymB) matched the Lee et al. (10) motif. We then checked if any of the genes that were differentially expressed in the *nolR*^+^ strain were located within 300 bp downstream of a predicted NolR motif. Cren et al. (11) NolR sites were present upstream of 20 of the 43 downregulated putative transcriptional units and four out of 28 of the upregulated putative transcriptional units (Table S3). Stringent Lee et al. (10) motifs were located upstream of only four differentially expressed genes: *nodD1*, *nodD2*, *nodA*, and the lipopolysaccharide (LPS) sulfotransferase gene *lpsS*, which was the most heavily downregulated gene in the *nolR*^+^ cultures.

We confirmed NolR binding to the *lpsS* upstream sequence by EMSA (Fig. 4B), suggesting that NolR may directly repress *lpsS* expression. As with the *nodD1* upstream probe, a high protein:probe ratio was required to show a shift. At higher protein:probe ratios, we observed a distinct second shift of the *lpsS* upstream probe, indicating that multiple NolR dimers may be binding within the region, although there is only a single complete predicted NolR site within the *lpsS* probe sequence (Table S2).

The high ratio of NolR protein to probe that was needed to produce shifts with both the *nodD1* and the *lpsS* upstream sequences may indicate relatively low affinity of NolR for the DNA or low stability of the complex *in vitro*. Since NolR binds DNA as a dimer (10), the addition of the His tag may limit effective dimerization. Alternatively, the purification process may have compromised NolR’s DNA binding capability.

## DISCUSSION

We conducted this study to determine why the antiphage activity of the Hna phage defense system varies so dramatically in two *S*. *meliloti* strains derived from the same parent. Our analysis revealed that this difference is due to a *nolR* mutation. The protective efficacy of the Hna system is sensitive to changes in its expression level, which is affected by the transcriptional regulator NolR. The presence of a functional copy of *nolR* increases *hna* expression and hence improves the bacterium’s phage defense ability. This is the first time that the NolR regulator has been implicated in the control of phage defense. Below, we first address members of the NolR regulon uncovered in this study and then discuss how *nolR* affects the Hna-dependent phage resistance phenotype.

Potential new members of the NolR regulon were identified based on their differential expression in *nolR*^+^ vs. Δ*nolR* strains. The presence of predicted NolR binding sites upstream of several of these genes (e.g., the predicted BioY-like biotin transporter gene *bioB* and the TadE/TadG family type IV pilus assembly gene *smc04280*) suggests that some may be direct targets of NolR regulation. In particular, we propose that the LPS sulfotransferase gene *lpsS* is regulated by NolR. In our RNA-seq experiment, *lpsS* was the most highly downregulated gene in the *nolR*^+^ strain, and we have demonstrated that the NolR protein binds upstream of *lpsS*, likely to a predicted NolR recognition site located 67 bp upstream of the start codon.

Expression of *lpsS* has previously been found to respond to the SyrA protein, likely via its interaction with the ExoS/ChvI two-component regulatory system, as well as to the NodD3 regulator (45, 46). LpsS expression is also downregulated in the presence of divalent cations (47). These studies of *lpsS* expression were conducted in an Rm1021 (i.e., *nolR*^–^) background, so they do not account for the effect of NolR on *lpsS* expression. The biological relevance of LPS sulfation in *S. meliloti* is not well understood (39), but considering the *lpsS* gene’s apparent direct regulation by NolR, as well as its response to other regulators of symbiosis and nodulation genes, precise control of this process may be important for symbiosis.

Our RNA-seq analysis also revealed surprising differential expression of multiple genes without predicted upstream binding sites. Four out of the eight methyl-accepting chemotaxis proteins encoded across the *S. meliloti* genome were downregulated in *nolR^+^* cells (*mcpV*, *mcpU*, *mcpW, *and *mcpT*) (48). We also observed increased expression of several distinct gene clusters involved in utilization of inositol (*iolCDEB*, *iolA, *and *iatPAibpA*) in *nolR^+^*cultures. *iolCDEB* and *iolA* encode genes for inositol catabolism, while the *iatPA* and *ibpA* cluster encodes a putative inositol transporter (49, 50). The *iolCDEB* genes have previously been found to be regulated by the ExoS/ChvI two-component system and are important for symbiotic competitiveness during nodule formation on alfalfa (49, 51).

Previous studies (12, 13) have relied on proteomic analysis to elucidate the *S. meliloti* NolR regulon: ours is the first to examine mRNA levels. There is little overlap between the NolR-regulated genes identified in any of the three studies of NolR targets (Table S7). Only three of the genes differentially expressed between strains in our RNA-seq experiment were reported by Chen et al. (13) as showing differential protein accumulation in a *nolR*^– ^vs. *nolR*^+^ Rm41 derivative strain: *smc02171*, *smc03131*, and *smc03135*. An earlier study of NolR regulation in *S. meliloti* reported N-terminus sequences of proteins differentially accumulated in *nolR*^+^ and *nolR*^–^ strains during late exponential/early stationary phase (12). We matched these sequences to their most likely protein counterparts in *S. meliloti* Rm1021 using blastp (task: blast-short) and found only three whose corresponding genes were differentially expressed in our RNA-seq results: *smc01242*, *smc02503*, and *smc04385*. Only two of these six genes (*smc02171* and *smc03131*) displayed a log-fold expression change >1 or < –1 in our analysis, and only *smc03131* and *smc03135* matched in the direction of regulation (up/down) between studies. We were also able to detect differences in *nod* gene expression, which were not detected in previous proteomic studies (12, 13). The large discrepancy between the RNA-seq and proteomic results may be partly attributable to the limitations inherent in the examination of 2D protein gels, as only a subset of proteins observed to be differentially accumulated could be identified, and relatively small differences in protein levels may have been overlooked. Differences in culture growth phase as well as genetic background (Rm41 vs. Rm1021) between experiments may also explain some of the variation between studies.

Given the wide variety of genes that respond to NolR, characterizing NolR as a global regulator seems apt (12). However, NolR remains strongly implicated in the control of symbiosis genes. In *meliloti* as well as in other *Sinorhizobium* species, NolR participates in a complex regulatory network that includes the NodD transcriptional regulators (NodD1, NodD2, and NodD3 in *meliloti*), as well as SyrM, SyrA, and the ExoS/ChvI two-component system (11, 15, 46). Together, these regulators control the expression of many genes involved in nodulation and the establishment of symbiosis (11, 15, 46, 51–53). We were intrigued to note that *hna* (*sma2245*) expression had previously been found to double upon overexpression of *nodD3*, another member of this regulatory network (46). There is no predicted NodD binding motif (*nod* box) upstream of *hna* (54, 55). The mirrored effects of NolR and NodD3 on *hna* expression cannot be explained by the regulation of NolR by NodD3 or vice versa, since NolR affects the Hna phage resistance phenotype even in a strain lacking pSymA. Instead, we suspect that *hna* expression is influenced by a common target of NolR and NodD3, which is encoded on either the chromosome or pSymB.

NolR’s regulation of *hna* is likely indirect: there is no predicted NolR binding site upstream of *hna*, and NolR does not bind to the *hna* upstream sequence *in vitro* (Fig. 4A). *hna* expression may be indirectly influenced by physiological differences between *nolR*^– ^vs. *nolR*^+^ cells. Alternatively, it is possible that *hna* expression is regulated by a regulator that is itself differentially expressed in the presence of *nolR*. Several genes encoding putative transcriptional regulators (other than *nodD1* and *nodD2*) are differentially expressed in the *nolR*^+^ strain, including the acid stress regulator gene *phrR*, *smeR* (*smc02866*), which encodes a regulator of the *smeAB* multidrug efflux pump genes, and *smc00878* (56, 57). *Smc00878* encodes a so-called “orphan” LuxR-like regulator that is not associated with its own acyl homoserine lactone synthase (58). Patankar and Gonzalez have proposed that Smc00878 may activate the expression of denitrification genes, as the *nap*, *nor*, and *nos* denitrification clusters were found to be downregulated in an *smc00878* mutant (59). Interestingly, while Smc00878 is downregulated in *nolR*^+^ cells compared to *nolR*^–^ cells, we see increased expression of the *nap* genes *napF*, *napD*, *napA*, *napB*, and *napC* in this strain. We also identified a putative NolR binding site 160 bp upstream of the *nap* operon.

The regulation of such apparently disparate genes by NolR may represent a cohesive response to specific environmental conditions. Uncovering the physiological signal(s) that govern *nolR* expression could help explain its regulatory function. NolR may play a role in responding to environmental stressors, as its expression decreases in response to nutrient limitation, pH shock, and low oxygen conditions (13). A *nolR^+^* culture was also found to be more tolerant to heat shock and showed improved survival in nutrient-limited conditions compared to a *nolR^–^* strain (13). In addition, the *nolR* promoter has been reported to bind the LuxR-like quorum sensing regulator ExpR (60), and *nolR* expression may be influenced by culture growth phase (13), although there is currently no direct evidence for quorum sensing regulation of *nolR* (13, 60, 61).

We were surprised that Hna’s antiphage activity was so strongly influenced by a mere doubling of its transcription, since *hna* appears to be expressed even in the absence of a functional *nolR*. In our β-glucuronidase assays with *nolR*^–^ strains, the *hna-gusA* fusion displayed promoter activity significantly above background levels and only approximately two times lower than a fusion to the housekeeping gene *gyrB* encoding DNA gyrase subunit B (Fig. 2A; Fig. S3). We also compared the mean counts of RNA-seq reads mapped to each coding sequence in our Δ*nolR* strain (after normalizing for library size and CDS length, see Table S8). The number of reads mapped per kb of coding sequence for *hna* was equivalent to the reads/kb counted for the essential thymidylate kinase gene *tmk* (62, 63), and only three times fewer reads/kb were mapped to the *hna* coding sequence than to the *lexA* CDS (3690 vs 11229).

The protective phenotype’s sensitivity to expression changes suggests that a threshold concentration of Hna in the cell is required for effective phage defense. Multiple Hna proteins may be required to completely counteract phage replication (a “many soldiers” model). Alternatively, if Hna-mediated defense requires rapid detection of phage infection before some crucial point in the phage life cycle, then a higher concentration of Hna in the cell improves the chances of intercepting phage replication, even if the deciding defensive action may be carried out by a single protein (a “many sentinels” model). As Hna is present in *nolR*^–^ cells, the phage must be able to escape or overwhelm the system in a sufficient percentage of infections to generate plaques. Our findings highlight the importance of considering genetic background and its effect on gene expression when discussing the efficacy of phage defense systems; even an apparently strong system may not confer a strong benefit in all strains.

## SUPPLEMENTAL MATERIAL

Supplemental material jb.00182-25-s0001.pdf Fig. S1 to S3, Tables S2 and S5, Note S1, and supplemental references.

Table S1 jb.00182-25-s0002.xlsx Strains and plasmids used in this study.

Table S3 jb.00182-25-s0003.xlsx Differential expression analysis results.

Table S4 jb.00182-25-s0004.xlsx Differential expression analysis results for RNA-seq pilot experiment (see Note S1).

Table S6 jb.00182-25-s0005.xlsx Positions of predicted NolR binding sites identified in the *S. meliloti* genome.

Table S7 jb.00182-25-s0006.xlsx Comparisons between differentially accumulated proteins observed in the Chen et al. 2000 and Chen et al. 2005 *nolR* studies and differentially expressed genes identified in this study.

Table S8 jb.00182-25-s0007.xlsx Normalized RNA-seq read counts for each CDS feature per kb of CDS length.

ASM does not own the copyrights to Supplemental Material that may be linked to, or accessed through, an article. The authors have granted ASM a non-exclusive, world-wide license to publish the Supplemental Material files. Please contact the corresponding author directly for reuse.
