# Supplementary material for: Unexpected modulation of Hna phage defense activity by the symbiotic regulator NolR
Source: J Bacteriol. 2025 Aug 18;207(9):e00182-25. doi: 10.1128/jb.00182-25 (PMC12445080; doi:10.1128/jb.00182-25)
Supplement: Supplemental material — Fig. S1 to S3, Tables S2 and S5, Note S1, and supplemental references. [file jb.00182-25-s0001.pdf]

Unexpected modulation of Hna phage defence activity by the symbiotic regulator NolR – Supplemental material

Leah M. Sather, Niousha Fazeli, Jason V. S. Kearsley, Kathryn Jones, and Turlough M. Finan

Table S2: Sequences of EMSA probes. Predicted NolR binding sites are underlined. Nucleotides within the gene's start codon are shown in bold.

Probe	Sequence
nodD1 upstream (241 bp)	TCAAAGCACGATCGAGAGTGGTGTGTACCCGGCAAGTTACACCGGG<br>GACGCCAACCGTAGCGCTGGACCGTTGGTCGAGCGCTTAGATGACG<br>GCTCTAAATTCGGACCCATCCTGTACTTGTGAAACTTTTCTCGCGC<br>CGCACCTTGATTCCATTAAC<u>TTCAGGGTTCTCTAAT</u>AGGACTCTGC<br>AAGATTGGTAAAATTGATTGTTTGGATAACGATCATCTGCGATATG<br>GATGCCGCAC**A**
hna upstream (283 bp)	GGGAACGAGTAACCGACATTGGCCGCCATCGATGCGGTGCTTGCCC<br>GATGGGAGGCGGCGATTGAGGACGCAAAGGCGCCGGGCCGCACGCG<br>AGAAGGATCCGCGATAGTGCCGTGGCGGTCTGGATCGCATCGAGGA<br>GTCGAGGTACTCCAGCGTCCCGCCAGAATACCAATTGGTTTGCGCG<br>TGGTCAGTTTTCGCTGGAACTATACTTTACGCCACCACTTGAAAAC<br>GAGCGTCGACTCCCATACCCATCCCTTAGATTGTAGTCGGGGGATA<br>CATA**GTG**
lpsS upstream (210 bp)	GCAATGCTGCAATAATGTCGCCGCTCTTCATCTGATACGACAATAC<br>ACACGCATGTGTATTCGTCCAATGAATTTGTCAAGCCAATCTAAGG<br>CGGCTTTCTCGGATGGTCTCGAACCGTCGGATC<u>ATCAGCTTGTGCT</u><br><u>AAA</u>AAAGCCAATATTCATCTCTAACGACCGCCTGGCTGGGACTTCG<br>AAGCGAGTTCGGACGGAGAGTTC**ATG**

Table S5: Phage 5A plaque counts when plated on *nolR*$^{+}$ and *nolR*$^{-}$ strains with and without *hna* provided on a plasmid under the P$_{tac}$ promoter. Strains were grown in the absence of inducer (IPTG).

Strain	Phage dilution plated	Plaques
RmP6132 (RmP110 Δ*nolR* with *hna*-His plasmid)	10^{-2}	0
	10^{-3}	0
RmP6133 (RmP110 *nolR*$^{+}$ with *hna*-His plasmid)	10^{-2}	0
RmP6135 (RmP110 Δ*nolR* with empty pTH1227)	10^{-8}	70
RmP6136 (RmP110 *nolR*$^{+}$ with empty pTH1227)	10^{-2}	0

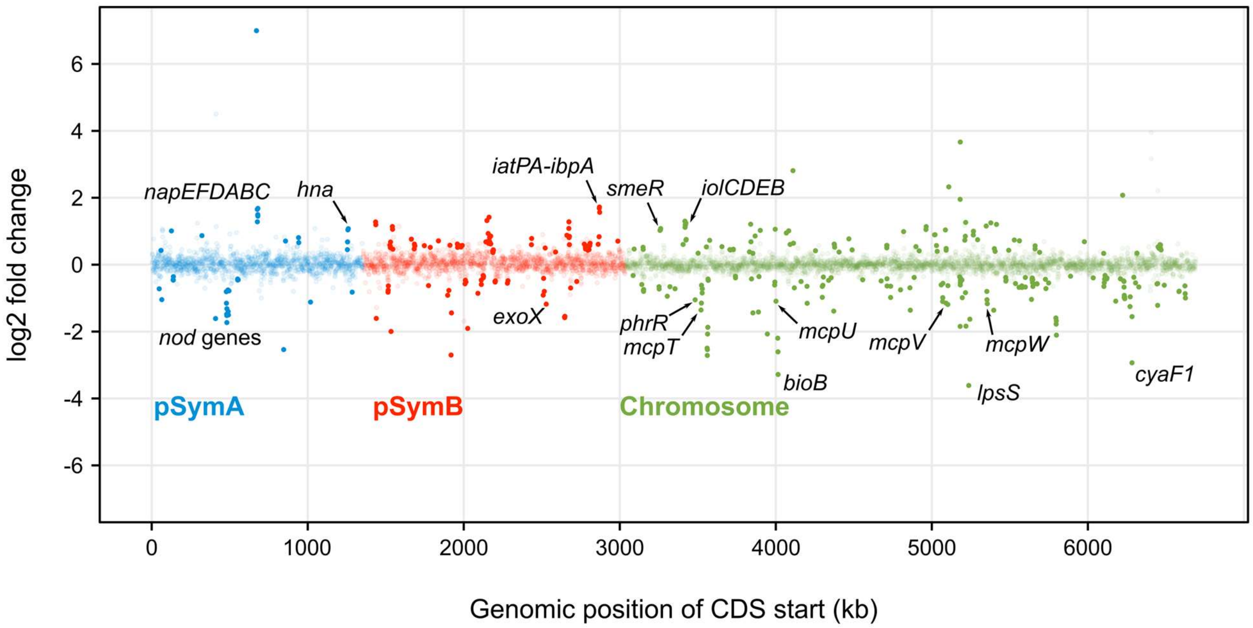

Figure S1: Differential expression of genes in the *nolR*$^{+}$ strain relative to the Δ*nolR* strain across the *S. meliloti* genome. Figure shows results of differential expression analysis of RNA-seq data, with each point representing a coding sequence (CDS). Genes demonstrating significant changes in expression between strains (adjusted *p*-value < 0.05) are shown as solid points, while those with *p*-values > 0.05 are transparent. Genes are colour-coded based on replicon (blue = pSymA, red = pSymB, green = chromosome). Selected genes are labeled.

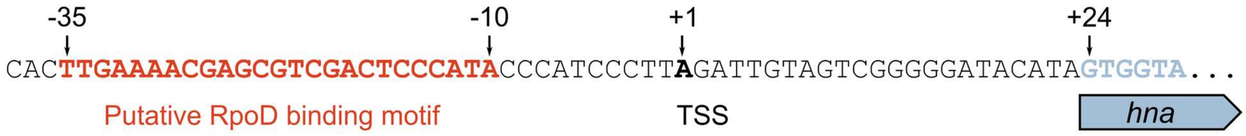

Figure S2: Predicted *hna* promoter region. The *hna* transcriptional start site (TSS; shown in bold) was identified through RNA-seq experiments by both Sallet et al. (13) (strain Rm2011) and Schlüter et al. (14) (strain Rm1021) and is designated as position +1. The putative RpoD-like binding motif reported in the Sallet et al. (13) and Schlüter et al. (14) studies is shown in red. The start of the *hna* coding sequence is shown in blue.

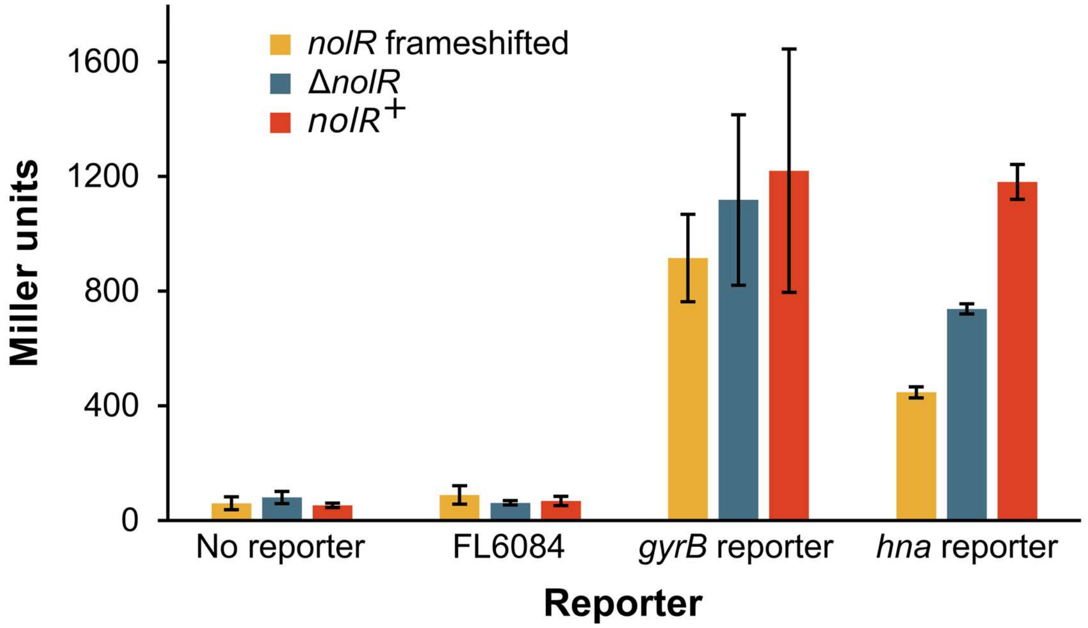

Figure S3: Beta-glucuronidase (GusA) activity from a *gyrB* promoter fusion compared to the *hna* promoter fusion. Results are from the same experiment depicted in Figure 2A, with the addition of results from *gyrB* reporter (FL5430) strains (omitted from Figure 2A for simplicity). Bars represent the means of three biological replicates. Error bars indicate means +/- SD.

Note S1: Description of the pilot RNA-seq experiment.

An additional RNA-seq experiment was performed with strains RmP6064 (RmP110 Δ*nolR* φ RmP4321), which carries an integrated copy of the empty pTH1937 vector at *hypRE* on pSymB, and RmP6065, which contains two copies of *nolR* at the native *nolR* site on the chromosome. RmP6065 was created by transduction of Nm^R from a strain in which pTH3546 (pTH1937 carrying a copy of *nolR*) was mistakenly integrated at *nolR* in a *nolR*$^+$ (Rm5000) background. Cultures were grown in duplicate. RNA extraction, sequencing, and analysis was conducted as described in Materials and Methods.

References for supplemental material

1. Finan T, Hartweig E, LeMieux K, Bergman K, Walker G, Signer E. 1984. General transduction in *Rhizobium meliloti*. J Bacteriol 159:120–124.
2. Meade HM, Long SR, Ruvkun GB, Brown SE, Ausubel F. 1982. Physical and genetic characterization of symbiotic and auxotrophic mutants of *Rhizobium meliloti* induced by transposon Tn5 mutagenesis. J Bacteriol 149:114–122.
3. Yuan Z-C, Zaheer R, Finan TM. 2006. Regulation and properties of PstSCAB, a high-affinity, high-velocity phosphate transport system of *Sinorhizobium meliloti*. J Bacteriol 188:1089–1102.
4. Geddes BA, Kearsley JV, Huang J, Zamani M, Muhammed Z, Sather L, Panchal AK, diCenzo GC, Finan TM. 2021. Minimal gene set from *Sinorhizobium* (*Ensifer*) *meliloti* pSymA required for efficient symbiosis with *Medicago.* Proc Natl Acad Sci USA 118:e2018015118.
5. Sather LM, Zamani M, Muhammed Z, Kearsley JV, Fisher GT, Jones KM, Finan TM. 2023. A broadly distributed predicted helicase/nuclease confers phage resistance via abortive infection. Cell Host Microbe 31:343–355.
6. Cowie A, Cheng J, Sibley CD, Fong Y, Zaheer R, Patten CL, Morton RM, Golding GB, Finan TM. 2006. An integrated approach to functional genomics: construction of a novel reporter gene fusion library for *Sinorhizobium meliloti*. Appl Environ Microbiol 72:7156–7167.
7. Finan TM, Kunkel B, De Vos GF, Signer ER. 1986. Second symbiotic megaplasmid in *Rhizobium meliloti* carrying exopolysaccharide and thiamine synthesis genes. J Bacteriol 167:66–72.
8. Leigh JA, Signer ER, Walker GC. 1985. Exopolysaccharide-deficient mutants of *Rhizobium meliloti* that form ineffective nodules. Proc Natl Acad Sci USA 82:6231–6235.
9. Milunovic B, diCenzo GC, Morton RA, Finan TM. 2014. Cell growth inhibition upon deletion of four toxin-antitoxin loci from the megaplasmids of *Sinorhizobium meliloti*. J Bacteriol 196:811–824.
10. Zhang Y, Aono T, Poole P, Finan TM. 2012. NAD (P)+-malic enzyme mutants of *Sinorhizobium* sp. strain NGR234, but not *Azorhizobium caulinodans* ORS571, maintain symbiotic N_2 fixation capabilities. Appl Environ Microbiol 78:2803–2812.
11. Schuster LA, Reisch CR. 2021. A plasmid toolbox for controlled gene expression across the Proteobacteria. Nucleic Acids Res 49:7189–7202.

12. Cheng J, Sibley CD, Zaheer R, Finan TM. 2007. A *Sinorhizobium meliloti minE* mutant has an altered morphology and exhibits defects in legume symbiosis. Microbiology 153:375–387.

13. Sallet E, Roux B, Sauviac L, Jardinaud M-F, Carrere S, Faraut T, de Carvalho-Niebel F, Gouzy J, Gamas P, Capela D, Bruand C, Schiex T. 2013. Next-generation annotation of prokaryotic genomes with EuGene-P: application to *Sinorhizobium meliloti* 2011. DNA Res 20:339–354.

14. Schlüter J-P, Reinkensmeier J, Barnett MJ, Lang C, Krol E, Giegerich R, Long SR, Becker A. 2013. Global mapping of transcription start sites and promoter motifs in the symbiotic α -proteobacterium *Sinorhizobium meliloti* 1021. BMC Genomics 14:156.
